# Expression analysis on 14-3-3 proteins in regenerative liver following partial hepatectomy

**DOI:** 10.1590/1678-4685-GMB-2017-0029

**Published:** 2017-11-06

**Authors:** Deming Xue, Yang Xue, Zhipeng Niu, Xueqiang Guo, Cunshuan Xu

**Affiliations:** 1 College of Life Science, Henan Normal University, Xinxiang, Henan, China; 2 Key Laboratory for Cell Differentiation Regulation, Xinxiang, Henan, China; 3 Academy of Fine Arts, Henan Normal University, Xinxiang, Henan, China

**Keywords:** liver regeneration, 14-3-3 proteins, western blotting

## Abstract

14-3-3 proteins play a vital part in the regulation of cell cycle and apoptosis as signaling integration points. During liver regeneration, the quiescent hepatocytes go through hypertrophy and proliferation to restore liver weight. Therefore, we speculated that 14-3-3 proteins regulate the progression of liver regeneration. In this study, we analyzed the expression patterns of 14-3-3 proteins during liver regeneration of rat to provide an insight into the regenerative mechanism using western blotting. Only four isoforms (γ, ε, σ and τ/θ) of the 14-3-3 proteins were expressed in regenerative liver after partial hepatectomy (PH). The dual effects, the significant down-regulation of 14-3-3ε and the significant up-regulation of 14-3-3τ/θ at 2 h after PH, might play particularly important roles in S-phase entry. The significant peaks of 14-3-3σ at 30 h and of ε and τ/θ at 24 h might be closely related not only to the G_2_/M transition but also to the size of hepatocytes. Possibly, the peak of 14-3-3ε expression seen at 168 h plays critical roles in the termination of liver regeneration by inhibiting cellular proliferation.

## Introduction

Liver has a remarkable capability of regenerating following different kinds of damage (Cienfuegos *et al.*, 2014). Liver is composed of numerous types of cells including hepatocytes, sinusoidal endothelial cells, stellate cells, Kupffer-Browicz cells, and biliary epithelial cells (Kang *et al.*, 2012). Nevertheless, hepatocytes, which account for approximately 80% of the liver mass and around 70% of liver cells, perform most of the functions of metabolism and synthesis (Si-Tayeb *et al.*, 2010). In seriously injured liver with damaged proliferation of hepatocytes, progenitor cells are thought to be helpful for regeneration through proliferation and differentiation (Alison *et al.*, 2009). In contrast, regeneration following partial hepatectomy (PH) does not need such a progenitor cell. The remnant liver undergoes compensatory hypertrophy and recovers the initial liver weight within approximately a week in rodents (Michalopoulos, 2007). The multi-lobe structure of the liver permits resecting one or more lobes to obtain various degrees of loss of liver weight. Because resecting the liver lobes does not injure the remnant tissue, PH is thought to be a very good experimental model for research into the regenerative mechanisms of this tissue. (Miyaoka and Miyajima, 2013).

The family of 14-3-3 proteins comprises a group of highly homologous acidic proteins expressed in all eukaryotic organisms. This family is made up of seven isoforms in human and rodent tissues (β/α, γ, ζ/δ, σ, ε, η, τ/θ) and plays an important role in the regulation of many cellular processes, including cell cycle, cell differentiation, apoptosis, DNA repair, motility and adhesion. 14-3-3 proteins function as phosphoserine/phosphothreonine-binding modules that take part in phospho-dependent protein-protein interactions (Gardino and Yaffe, 2011; Wu *et al.*, 2015). Their expression is tissue-specific.

Cell division and apoptosis take place during hepatic regeneration following 2/3 PH (Sakamoto *et al.*, 1999). Therefore, the 14-3-3 protein family may be closely linked to hepatic regeneration. Previously we reported the expression patterns of 14-3-3 mRNAs in regenerative liver following 2/3 PH by real-time qRT-PCR (Xue *et al.*, 2015). In the current study, we further analyzed the expression patterns of 14-3-3 proteins by western blotting.

## Materials and Methods

### Animals and PH model

Sprague Dawley male rats (200 ± 10 g) were obtained from the Animal Center of Henan Normal University. The rats were permitted *ad libitum* access to food and water. For the PH model, rats were anaesthetized by ether inhalation, and the left lateral and median lobes, which account for two-thirds of the total liver weight, were resected (Stolz *et al.*, 1999). The remaining liver lobes were obtained at 0, 2, 6, 12, 24, 30, 36, 72, 120 and 168 h after PH. All samples of liver were quickly frozen in liquid nitrogen, and then stored at -80 °C. All rats were placed in the facility of Animal Center of Henan Normal University, and all procedures were performed according to the Animal Protection Law of China.

### Protein extraction and western blotting

The regenerating liver tissues stored in liquid nitrogen were ground into fine powder and then suspended in extraction buffer (7 M urea, 2 M thiourea, 4% CHAPS). Next, the suspension was vortex-mixed for 1 h at 4 °C, and subsequently centrifuged at 20,000 x *g* for 1 h at 4 °C. The supernatants were collected and stored at –80 °C for further use. The protein concentration was assessed with the commercial RC DC^TM^ Protein Assay Kit according to the manufacturer’s instructions (BIO-RAD, USA). Protein samples, 50 μg, were separated by electrophoresis on 12% SDS/PAGE gels and subsequently electrophoretically transferred to polyvinylidene difluoride membranes (Millipore). The membranes were blocked with 5% non-fat milk, washed, and subsequently probed with antibodies against 14-3-3β/α, γ, ζ/δ, σ, ε, η, τ/θ (all 1:1000) and GAPDH (1:2000) (Sangon Biotech Co. Ltd., Shanghai, China)overnight at 4 °C. After being washed, the membranes were incubated with horseradish peroxidase-conjugated secondary antibodies (Sangon Biotech Co. Ltd., Shanghai, China), detected with an enhanced chemiluminescence detection kit (Boster Corporation, China) and then imaged in an ImageQuant LAS 4000 mini (GE Healthcare Bio-Sciences Corporation) system.

### Statistical analysis

Analysis of the western blots was carried out by a standard technique. The gray intensities of the bands were quantified by Image J software. The relative gray level value of the target band was normalized against that of the respective internal control GAPDH. Statistical analysis on protein expression was then carried out using SPSS version 16.0 (SPSS, Inc., Chicago, IL, USA) software. All data were reported as means ± standard deviation (n=3). Student’s *t*-tests were used for analyzing the expression difference between 0 h and the other time points after P, with p<0.05 indicating statistical significance.

## Results

**Figure 1:**
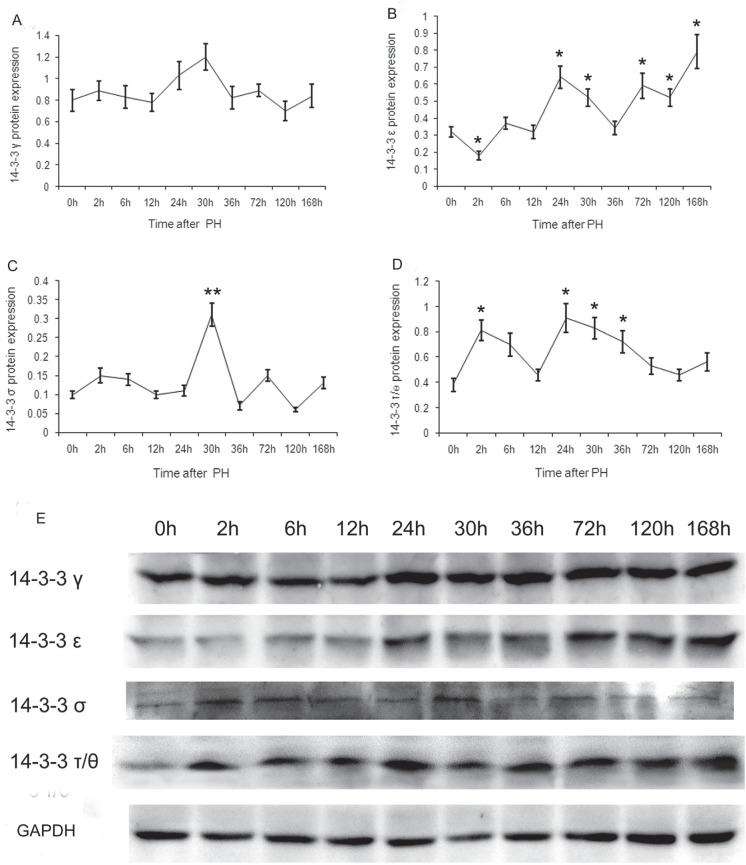
Expression of 14-3-3γ, ε, σ and τ/θ. Time curves for the expression (A) 14-3-3γ, (B) ε, (C) σ and (D) τ/θ at each time point after PH, analyzed by western blotting. The data are presented as means ± SD (n=3). Student’s *t*-tests were used for analyzing the expression difference between 0 h and the other time points after PH. *p < 0.05, **p < 0.01 *versus* 0 h. (E) Western blot detection of 14-3-3γ, ε, σ and τ/θ at the respective time points after PH. GAPDH was used as an internal control.

The expression patterns of the seven 14-3-3 protein isoforms in regenerative liver after PH were assessed by western blotting using an enhanced chemiluminescence detection kit. The results showed that only four (γ, ε, σ and τ/θ) of the 14-3-3 isoforms were expressed (Figure 1). When compared with 0 h, the expression of 14-3-3γ at the other time points showed wavy oscillations without significant difference (t < t_0.05(4)_=2.776, p > 0.05) (Figure 1A).

14-3-3ε expression, which showed a minimum at 2 h, was significantly down-regulated in comparison with that at 0 h (t=3.105>t_0.05(4)_=2.776, p<0.05). 14-3-3ε expression was significantly up-regulated at 24, 30, 72, 120 and 168 h (t=3.871, 2.970, 2.895, 2.970 and 3.899 > t_0.05(4)_ = 2.776, p<0.05). The transcript level at 168 h was the highest, and that at 30 h and 120 h was the lowest among these (Figure 1B).

14-3-3σ expression, with a peak at 30 h after PH, was significantly up-regulated in comparison with that at 0 h (t = 5.750 > t_0.01(4)_ = 4.601, p<0.01), whereas those at the other time points showed no significant difference, exhibiting wavy oscillations (t < t_0.05(4)_ = 2.776, p > 0.05) (Figure 1C).

When compared with 0 h after PH, the 14-3-3τ/θ expression at 2, 24, 30 and 36 h was significantly up-regulated (t = 3.948, 3.660, 3.952 and 2.860 > t_0.05(4)_ = 2.776, p<0.05). The transcript level at 24 and 36 h were the highest and lowest, respectively (Figure 1D).

## Discussion

Liver regeneration has been extensively investigated, but many essential mechanisms are still vague, for instance, the mechanisms of cell hypertrophy, cell division, and regulation of organ size (Miyaoka and Miyajima, 2013). For a long time, it has been thought that after 70% PH all remnant hepatocytes would undergo one or two rounds of cell division for recovery of the initial cell number and liver weight (Duncan *et al.*, 2009). Recent studies have reported that after 30% PH, the hepatocyte size of the remnant liver increases and recovers its initial weight, but the hepatocyte number does not change. In comparison, upon 70% PH, hepatocyte hypertrophy occurs for several hours following PH, before the cells proliferate. Although nearly all hepatocytes enter S-phase, only approximately half go through the cell division cycle and increase cell number. As a result, after 70% PH the liver recovers its original weight through both hypertrophy and proliferation of hepatocytes (Miyaoka *et al.*, 2012).

The sophisticated process of regeneration is divided into three different stages: an initial phase, a proliferative phase, and a terminative phase. In rats, within less than 15 min following PH, quiescent hepatocytes withdraw from the G_0_ phase and go into G_1_ phase. The first proliferation wave of hepatocytes after PH is simultaneous. A DNA synthesis peak was shown to occur between 22 and 24 hours, followed by the karyokinesis peak between 28 and 30 hours (Corlu and Loyer, 2012). The 14-3-3 proteins act on the G_1_/S and G_2_/M transitions by combining with cell cycle regulation proteins and regulating their function (Hermeking and Benzinger, 2006; Gardino and Yaffe, 2011), and therefore, they presumably play critical roles in the regulation of hepatic regeneration.

Successful activation of different cyclin-dependent kinases (CDKs) is required for passing through the cel1cycle. These kinases are regulated by transient binding of regulatory subunits, phosphorylation and dephosphorylation (Sherr and Roberts, 1995; Harper and Brooks, 2005).

The 14-3-3 proteins participate in the modulation of transition from G_1_ into S through a variety of mechanisms. They associate with and negatively modulate cell division cycle phosphatases (CDC25), which are also concerned with the regulation of CDK complexes that are extremely important for the transition from G_1_ to S. CDC25A, a key element for the entry into S phase by activating CDK2 through dephosphorylation, is inhibited by 14-3-3ε through cytoplasmic sequestration (Chen *et al.*, 2003). In addition, direct binding of 14-3-3σ to the kinases CDK2 and CDK4 negatively regulates S-phase entry (Laronga *et al.*, 2000). The 14-3-3 proteins can immediately bind to the CDK inhibitor p27 as well, which opposes p27-mediated G_1_ arrest. Following phosphorylation by the serine-threonine kinase AKT at Thr-198 and Thr-157, p27 associates with the 14-3-3β, γ, ζ, ε, η and τ isoforms, and does not function as CDK inhibitor because of its cytoplasmic sequestration (Fujita *et al.*, 2002; Liang *et al.*, 2002; Shin *et al.*, 2002), as the association hinders the binding of p27 to importin α (Sekimoto *et al.*, 2004). Through this mechanism, the 14-3-3 proteins may contribute to cell cycle progression. Furthermore, the p90 ribosomal protein S6 kinase also associates with and directly phosphorylates p27^KIP1^ on Thr-198, resulting in the binding to 14-3-3ε, η, σ, and τ isoforms (Fujita *et al.*, 2003). At 2 h after 2/3 PH, the significant down-regulation of 14-3-3ε and the significant up-regulation of 14-3-3τ may lead to the release of CDC25A from 14-3-3ε and the association of p27^KIP1^ with 14-3-3τ, respectively. These dual effects might play particularly important roles in S-phase entry.

14-3-3 proteins are also necessary for the transition from G_2_ into M (Ford *et al.*, 1994). In mammalian cells, a key step for mitotic entry is the activation of CDC2 kinase (Norbury *et al.*, 1991). During S phase, CDC2 activity is inhibited through phosphorylation by the MYT1/MIK1 and WEE1 kinases at Thr-14 and Tyr-15 (Parker and Piwnica-Worms, 1992; Liu *et al.*, 1997). CDC25C dephosphorylates CDC2 at Thr-14 and Tyr-15, which leads to CDC2 activation and initiates entry into M phase (Gautier *et al.*, 1991). Activating CDC25C is indispensable for promoting cell cycle progress, whereas its restraint is connected with the activation of the G_2_/M checkpoint (Donzelli and Draetta, 2003). CDC25C activity is suppressed following phosphorylation on the residue Ser-216, mediated by CHK1, CHK2 or C-TAK1. This occurs through subsequent association with 14-3-3 isoforms and sequestration in the cytoplasm. This causes the increase in CDC2 phosphorylation, leading to decreased activity and repression of entry into mitosis (Sanchez *et al.*, 1997; Peng *et al.*, 1998; Chaturvedi *et al.*, 1999). Seemingly, not all of the 14-3-3 isoforms are able to associate with and modulate CDC25C. CDC25C is able to interact with 14-3-3τ and ζ in lung adenocarcinoma A549 cells (Qi and Martinez, 2003). Only 14-3-3γ and ε among seven different 14-3-3 isoforms specifically associate with CDC25C in U2OS cells and thereby restrain CDC25C from inducing premature chromatinic condensation. In contrast, 14-3-3σ does not associate with CDC25C, but obstructs premature chromatinic condensation induced by the CDC25C mutant S216A and wild type CDC25C (Dalal *et al.*, 2004). This shows that 14-3-3σ regulates mitotic entry downstream of CDC25C.

CDC25B activity is also inhibited by binding to 14-3-3 isoforms, which blocks substrate access of CDK1-cyclinB complexes to the CDC25B catalytic site (Astuti and Gabrielli, 2011). CDC25B associates with different isoforms of the 14-3-3 family, and it was shown to interact with 14-3-3β, ζ, σ, ε and η *in vivo* and with 14-3-3β, ζ and η in a yeast two-hybrid test (Mils *et al.*, 2000; Uchida *et al.*, 2004).

According to the experimental results and the above discussion, we propose the following hypotheses. The significant peaks of 14-3-3ε expression, τ at 24 h and of σ at 30 h after PH are related to the regulation of not only the G_2_/M transition, but also the size of hepatocytes. They may play critical roles in preventing the remnant hepatocytes from prematurely entering into mitosis after PH. As a result, the size of the hepatocytes increases. It is reported that the terminative process in liver regeneration has not been adequately investigated, compared with the initiation (Miyaoka and Miyajima, 2013). The significant up-regulation of 14-3-3ε from 72 to 168 h after PH might be one of the important factors inhibiting the G_1_/S-transition by a direct association with CDC25A, and thus, would be key for the termination of hepatic regeneration.
